# Diagnostic Value of Contrast-Enhanced Ultrasound in Differentiating Malignant from Benign Small Renal Masses After CT/MRI

**DOI:** 10.3390/jcm13216478

**Published:** 2024-10-29

**Authors:** Fabrizio Urraro, Marco Piscopo, Nicoletta Giordano, Gaetano Maria Russo, Luigi Gallo, Simona Magliocchetti, Diego Sandro Giordano, Vittorio Patanè, Davide Arcaniolo, Immacolata Cozzolino, Valerio Nardone, Salvatore Cappabianca, Alfonso Reginelli

**Affiliations:** 1 Department of Precision Medicine, University of Campania “Luigi Vanvitelli”, 80138 Naples, Italy; fabrizio.urraro@unicampania.it (F.U.); patavittorio@gmail.com (V.P.); salvatore.cappabianca@unicampania.it (S.C.); alfonso.reginelli@unicampania.it (A.R.); 2 Urology Unit, Department of Woman, Child and General and Specialized Surgery, University of Campania “Luigi Vanvitelli”, 80138 Naples, Italy; 3 Pathology Unit, Mental and Ohysical Health and Preventive Medicine Department, University of Campania “Luigi Vanvitelli”, 80138 Naples, Italy

**Keywords:** contrast-enhanced ultrasound (CEUS), diagnostic performance, kidney imaging, malignant and benign differentiation, small renal masses (SRMs)

## Abstract

**Background**: The aim of this study was to assess the diagnostic performance of contrast-enhanced ultrasound (CEUS) in characterizing small renal masses (SRMs) measuring less than 3 cm and in distinguishing between malignant and benign SRMs. **Methods**: A retrospective study was conducted between January 2022 and January 2023 at the Radiology Department of (Anonymized data), with a total of 43 patients assessed via CT and MRI scans, which were subsequently studied by experienced radiologists who were blinded to the pathology results. The CEUS findings were then compared with histopathological examination outcomes or follow-up imaging results. **Results**: The study results revealed a notably high level of diagnostic accuracy, with sensitivity at 0.875, specificity at 0.94, positive predictive value at 0.95, and negative predictive value at 0.86 for characterizing SRMs. Spearman rank correlation analysis substantiated a robust positive linear correlation between the CEUS findings and biopsy results (r = 0.972). **Conclusions**: These findings underscore the potential utility of CEUS as a valuable tool for discriminating between malignant and benign SRMs, carrying significant implications for clinical decision-making and leading to improved patient outcomes. However, larger validation studies are imperative to establish its role in routine clinical practice and to address potential limitations.

## 1. Introduction

Contrast-enhanced ultrasound (CEUS) has emerged as a significant imaging modality in the realm of radiology, providing real-time visualization and improved characterization of diverse organ systems [1,2]. Its utilization for noncardiac purposes has experienced a steady global growth trend [3,4]. Although categorized as “off-label”, renal applications experienced rapid emergence in the European context [5]. In the context of small renal masses (SRMs), CEUS has demonstrated its potential for delivering precise diagnoses and facilitating treatment determinations [6].

Small renal masses (SRMs) are defined as kidney lesions that are enhanced with contrast and have a maximum diameter of 4 cm or less [7,8,9,10]. These lesions often align with stage T1a renal cell carcinomas. The global detection of small, asymptomatic renal lesions, including cancers, has risen in recent years, largely attributed to the expanded utilization of cross-sectional imaging [7,11]. Currently, a significant proportion (50–61%) of all renal tumors are discovered incidentally, marking a stark increase compared to the 13% reported in the 1970s [4,12,13].

SRMs pose a diagnostic conundrum owing to their malignant potential. They constitute a significant portion (48–66%) of RCC diagnoses [14]. Remarkably, a substantial majority (79–84%) of SRMs are identified prior to the onset of genitourinary symptoms [15,16,17]. Despite a decreasing mean tumor size in recent years, numerous studies highlight the significance of this variable as a crucial prognostic factor for RCC [18]. In fact, it has influenced the latest revisions in RCC staging and treatment approaches [14].

Within this category, various benign tumors are identified in 12.8% to 17.3% of cases [19]. These benign lesions include oncocytoma (53%), angiomyolipoma (22%), and atypical cyst (10%), as well as other benign conditions like leiomyoma, xanthogranulomatous pyelonephritis, and focal infarction (13%). The likelihood of malignancy typically increases as the size of SRMs grows larger [20]. Although the mean size of incidental renal tumors is 3.7 cm (with a median of 3 and a range from 0.8 to 12 cm), it is important to note that tumors larger than 4 cm can also be discovered incidentally [16]. Incidental diagnoses occur in 82.4%, 78.9%, and 56.7% of renal masses measuring 1–4 cm, 4–6 cm, and over 6 cm, respectively [14]. While it has been observed that most cases of RCC smaller than 7 cm are incidentally found, tumors larger than 7 cm tend to be symptomatic. However, this distinction should not be universally applied as there are exceptions [21]. The timely and precise diagnosis of small renal lesions is imperative for formulating appropriate patient management strategies and optimizing clinical outcomes [22]. The promise of CEUS in renal imaging became apparent through pre-market animal investigations and the subsequent development of specialized contrast-specific imaging modes [23,24]. Contrast-enhanced ultrasound (CEUS) is an emerging technology designed to overcome some of the limitations of standard grayscale and Doppler ultrasound methods in detecting vascularization within soft tissues. Ultrasound contrast agents administered intravenously comprise small particles: gas-filled cores enclosed within biodegradable shells. These microbubbles, roughly the size of red blood cells, exhibit non-linear resonance under ultrasound waves. The distinct signals emitted by microbubbles can be distinguished from the surrounding tissues, enabling the specific identification of blood flow within perfused tissues. CEUS offers a reliable means to confirm simple cysts by their lack of enhancement and may assist in characterizing solid renal lesions by discerning differences in perfusion between the lesion and renal cortex [25,26]. Current applications are firmly established, and guidelines were published by the European Federation of Societies for Ultrasound in Medicine and Biology in 2012, updated in 2018. According to a prediction from these guidelines, the use of CEUS in renal applications is expected to increase further, improving patient outcomes and reducing healthcare costs [27].

CEUS is increasingly utilized, without regard to renal function, as a diagnostic tool for characterizing renal lesions identified either via ultrasonography or incidentally during computed tomography (CT) or magnetic resonance (MR) scans conducted for alternative clinical indications [28]. CEUS demonstrates superior sensitivity compared to contrast-enhanced CT and, at the very least, is on par with contrast-enhanced MR imaging in the detection of subtle perfusion irregularities [29,30]. Primarily, CEUS offers augmented visualization and characterization capabilities concerning renal masses, thereby enhancing the capacity to discern between benign and malignant lesions. By administering intravenous microbubble contrast agents, CEUS facilitates the evaluation of vascularity and perfusion within the renal mass, affording a real-time appraisal of blood flow dynamics that, in turn, furnishes crucial insights for lesion characterization [27,31,32]. Furthermore, CEUS offers the advantages of cost-effectiveness, expeditious execution, and exemption from the necessity for prior laboratory investigations, rendering it well-suited for bedside assessments. In contrast to MRI, CEUS frequently proves effective in assessing renal and tumor perfusion even in cases involving non-cooperative patients [33]. CEUS has assumed a pivotal role within the realm of diagnosing and managing diverse renal pathologies, encompassing renal masses, chronic kidney disease, and renal artery stenosis [34]. Furthermore, CEUS serves as a valuable modality for the evaluation of vascular anomalies within the kidney, notably renal artery stenosis, thereby furnishing critical insights essential for an informed treatment stratagem [35,36]. In the realm of medical practice, the precise identification and comprehensive characterization of small renal masses (SRMs) stand as a pivotal cornerstone for the clinical-surgical care of patients.

The aim of this study was to evaluate the potential role of contrast-enhanced ultrasound for characterizing small renal masses, whose examination via other radiologic techniques (e.g., CT and/or MRI) was not conclusive. Presented herein is a retrospective study encompassing cases observed from January 2022 to January 2023 at the Radiology Department of (Anonymized data), focusing on patients with SRMs that remained indeterminate via conventional CT or MRI examinations. Drawing upon our institutional experience, we underscore CEUS as an indispensable tool for the comprehensive characterization of SRMs and for its crucial role in guiding clinical and surgical interventions.

## 2. Materials and Methods

This retrospective study was conducted at a single medical center. The study’s protocol was approved by the local ethics committee at the (Anonymized data), Prot. 158/i/2022. Exemption from the requirement for patient informed consent was also obtained. The case cohort comprised 43 patients diagnosed with SRMs at (Anonymized data) between January 2022 and July 2023.

### 2.1. Patient Criteria and Diagnostic Route

Between January 2022 and July 2023, a total of 43 consecutive patients underwent CEUS after being diagnosed with renal masses smaller than 3 cm that were previously uncharacterized via contrast-enhanced CT and/or contrast-enhanced MR. In order to qualify for participation, individuals were required to fulfill the following criteria: being an adult aged 18 or older, having SRMs detected on initial imaging studies, being suitable for surgical biopsy, maintaining good health or having well-controlled chronic conditions managed with medication, having confirmation through fine-needle aspiration (FNA) or biopsy as well as surgical resection, and expressing willingness to take part in the study. Exclusion criteria included severe comorbidities leading to a life expectancy of less than 1 year, pregnancy, acute or chronic conditions impeding study involvement, or refusal to provide informed consent. Following multidisciplinary consultations involving urologists, surgeons, oncologists, and radiologists, CEUS imaging was performed alongside the routine CECT and CEMRI scans to provide a comprehensive evaluation of the small renal masses. The CEUS studies were conducted by a single radiologist at our institution, the Radiology Department of (Anonymized data). To minimize bias, the imaging studies, including CEUS, CECT, and CEMRI, were interpreted by experienced radiologists who were blinded to the pathology results. Blinding the radiologists to the final diagnosis helped reduce potential interpretation bias and ensured a more objective assessment of the imaging findings.

### 2.2. CEUS Assessment

The examinations were conducted at frequencies ranging from 1 to 6 MHz with convex array transducers. A 1.6 mL bolus of ultrasound contrast agent (SonoVue^®^, Bracco Imaging, Milan, Italy) was administered to all the patients, with a very low mechanical index (MI < 0.1) used to prevent early microbubble destruction. Penetration depth during the CEUS was adjusted by the investigator to clearly identify the target lesion and the entire kidney. Baseline B-mode ultrasound and CEUS (for qualitative assessment of contrast enhancement patterns) were performed by a single highly experienced radiologist with over 10 years of experience in CEUS.

**Figure 1:**
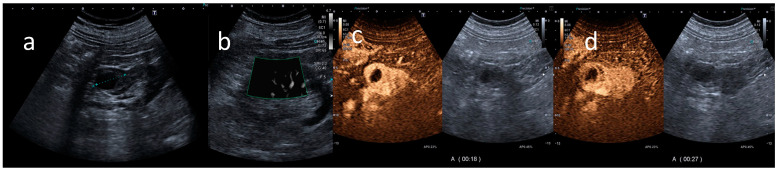
CEUS findings. B-mode ultrasound shows a complex cystic lesion irregularly thickened (**a**), with intravascular signal at SMI (**b**). In the arterial phase, after using a contrast agent (Sonovue), the contrast-enhanced ultrasound shows a marked enhancement (**c**) and a rapid wash-out (**d**) of the wall and of solid nodular components of the wall, with an un-enhanced central core of necrosis. These findings were assessed by the radiologist as positive findings for malignant small renal mass. Final pathologic assessment confirmed this assessment, as it was diagnosed as a renal cell carcinoma with foci of necrosis.

All the CEUS examinations were performed using a Canon Aplio a550 ultrasound machine (Canon Europe, Amstelveen, The Netherlands). Gray-scale B-mode ultrasound was employed for kidney lesion detection, kidney size assessment, and evaluation of echogenicity and homogeneity using a convex array transducer. The kidney was routinely examined in modified longitudinal and transverse planes and, if necessary, in deep inspiration and with optimized scanning positions. The CEUS examinations were performed during clinical routines using high-end ultrasound systems equipped with the most up-to-date CEUS-specific protocols available at the time of the examination (Figure 1).

### 2.3. Data Collection and Radiologic Assessment

Imaging characteristics were assessed retrospectively using static and dynamic anonymized images through the institutional Picture Archival and Communication System (PACS). To document findings digitally, short video clips of the cortical phase, the parenchymal/nephrogenic phase, and the late phase (>120 s post-injection) were recorded for each examination. The radiologists evaluated the imaging characteristics of the renal masses, including size, shape, enhancement pattern, and presence of necrosis or calcifications, among other features. The study analyzed the CEUS findings, including the enhancement patterns, vascularity, and morphological characteristics of the renal lesions. The results were compared with the final diagnoses obtained through histopathological examination or follow-up imaging studies.

All the identified lesions were categorized into two groups: cystic and solid lesions. Cystic lesions were assessed based on the Bosniak classification. Solid lesions were considered potentially malignant unless they displayed clear signs of being either inflammatory or ischemic in nature. Lesions that were deemed benign in the CEUS examination were designated as “definitely benign” if there were no indications of malignancy in the initial imaging. Histologically confirmed lesions were definitively categorized as either malignant or benign. For lesions that did not exhibit clear malignancy characteristics (classified as Bosniak IIF), a follow-up was initiated. These lesions were assigned to specific categories based on their imaging characteristics during subsequent follow-up assessments. To definitively label a lesion as malignant, either histological confirmation was required or it needed to exhibit strong malignancy indicators, such as clear interval growth, invasion of surrounding structures, or other evident tumor manifestations, via at least two different imaging modalities. The sensitivity, specificity, positive predictive value (PPV), and negative predictive value (NPV) of CEUS in characterizing small renal lesions were calculated to determine its diagnostic accuracy.

### 2.4. Statistical Analysis

In this study, the reliability of CEUS in distinguishing between benign and malignant small renal lesions was examined. Continuous variables were expressed using mean, standard deviation, median, percentiles, and symmetry; discrete and qualitative variables were represented by mode and percentages. Statistical analyses were performed using SPSS IBM. Sensitivity, specificity, positive predictive value (PPV), negative predictive value (NPV), and area under the curve (AUC) were calculated. The reliability of the parameters between biopsy and benign tumor yes/no was assessed using ROC curve analysis, which is a graphical representation of the sensitivity and specificity of the prediction model. Having obtained highly significant results, to maximize diagnostic accuracy, the Spearman rank correlation coefficient was calculated between benign tumor yes/no and biopsy, with a significance level set at 0.01.

## 3. Results

Out of the 43 patients included in the study, 29 were men (67%) and 14 were women (33%), with ages ranging from 37 to 87 years (mean age 62.30 years, median 64 years). Each patient in the study presented with a single renal lesion for characterization with a mean tumor dimension of 23.5 mm (minimum 10 mm, maximum 30 mm). Among these patients, 18 had previously undergone ultrasound examinations without contrast, followed by gadolinium-enhanced MRI; 21 had undergone ultrasound without contrast, followed by contrast-enhanced CT with intravenous contrast; and 4 patients had undergone ultrasound without contrast, followed by both contrast-enhanced CT examinations and gadolinium-enhanced MRI.

Patients without suspected oncological lesions (22) underwent ultrasound follow-up every 3 months, while others underwent histopathological analysis confirming our CEUS findings through surgical excision (15) or fine-needle aspiration biopsy (7). The study population characteristics are summarized in Table 1 and Table 2.

**Figure 2:**
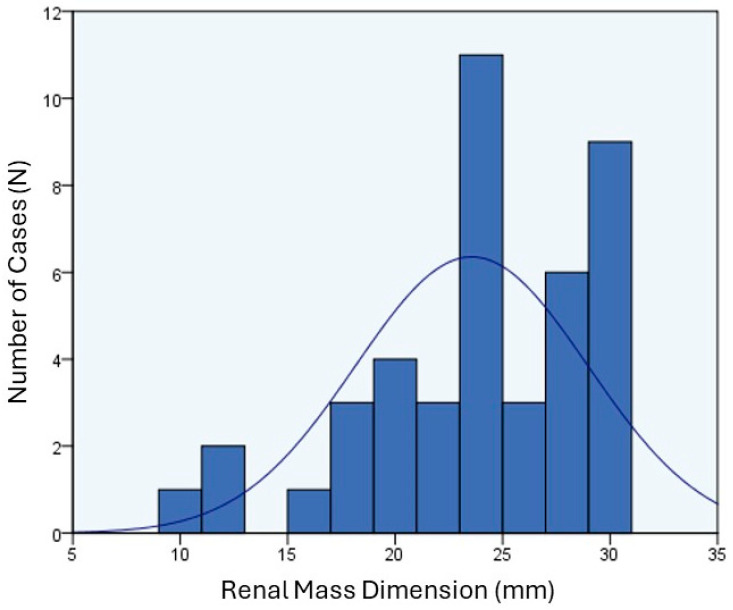
This chart illustrates statistical data, providing an overview of the central tendencies and dispersion within a dataset comprising 43 valid cases regarding SRM dimensions. The mean value is 23.56 mm, with a median of 24.00 mm. The standard deviation is approximately 5.400, indicating the degree of dispersion within the dataset, where a higher value suggests greater dispersion. The skewness is −0.860, indicating a slight leftward skew in the distribution of data. The kurtosis is 0.334, suggesting a slight flattening compared to a normal distribution. The range is 20, representing the difference between the maximum value (30) and the minimum value (10), thus highlighting the extreme values within the dataset. Additionally, the percentiles divide the data into equal parts, with the 25th percentile at 20.00, the 50th percentile (median) at 24.00, and the 75th percentile at 28.00.

Our radiologist encountered differentiation challenges between atypical angiomyolipoma and oncocytomas in only two cases. We specifically analyzed lesions measuring 30 mm or smaller. The chart illustrates the graphical distribution of lesion sizes in millimeters, with a mean of 23.56 mm, standard deviation of 5.40, and a median of 24 mm (Figure 2).

Among the 43 lesions included in our study, 9 were situated within the left mesorenal region, 10 within the right mesorenal region, 4 in the upper left pole area, 5 in the upper right pole area, 8 in the lower left pole area, and 7 in the lower right pole area. Out of 43 lesions, 25 (58%) were malignant lesions, and 18 (42%) were benign lesions. Specifically, the benign lesions included 1 subcapsular renal hematoma, 10 renal cysts classified as Bosniak IIF, 3 inflammatory lesions, 2 renal oncocytomas (ROs), and 2 renal angiomyolipomas (AMLs). The malignant lesions comprised 16 clear cell renal cell carcinomas (ccRCCs), 3 papillary renal cell carcinomas (PRCCs), 3 chromophobe renal cell carcinomas (ChRCCs), 1 eosinophilic solid and cystic renal cell carcinoma (ESC RCC), and 2 renal metastases from cutaneous melanoma.

**Figure 3:**
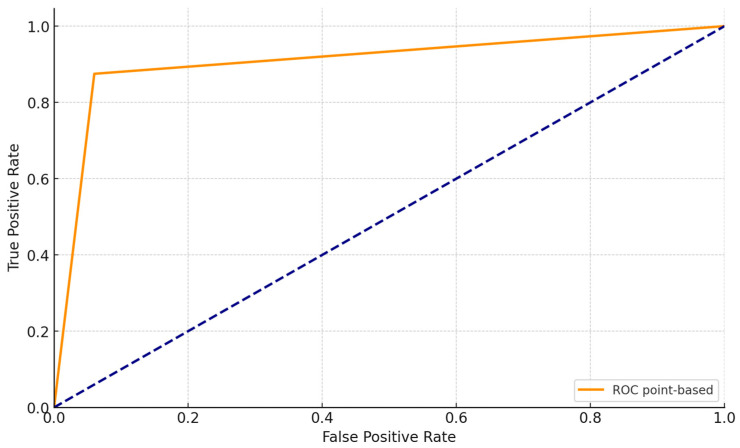
ROC curve comparing biopsy results (0 for healthy, 1 for ill) with benign outcomes (yes/no), demonstrating exceptional sensitivity (0.875).

Our analysis yielded significant findings despite the relatively small sample size of 43 subjects. Sensitivity, specificity, positive predictive value, and negative predictive value were computed comparing patients with the illness to non-ill patients (controls) based on the results of diagnostic tests, yielding the following results: sensitivity of 0.875, positive predictive value of 0.95, specificity of 0.94, and negative predictive value of 0.86. A ROC curve was generated comparing biopsy results (healthy individuals labeled as 0 and sick individuals as 1) and distinguishing between benign and non-benign cases. The test proved highly informative, particularly due to its high sensitivity (Figure 3).

**Figure 4:**
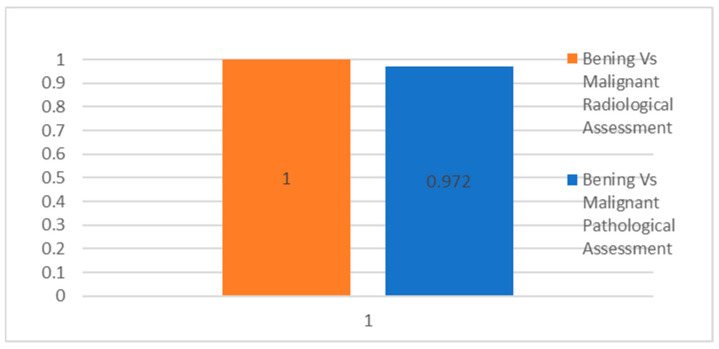
These data represent the Spearman correlation between “Benign/Malignant” and “Histology”. Spearman’s Rho: 1.000 (perfect correlation). Significance (2-tailed): 0.000 (highly significant). Sample size (N): 43. The correlation is statistically significant at the 0.01 level (2-tailed). It indicates a very strong positive relationship between the “Benign/Malignant” and “Histology” variables.

To validate the findings, the Spearman rank correlation was calculated indefinitely between the count of benign/malignant tumors and the biopsy outcome, using an alpha significance level of 0.01 (Figure 4). The resulting linear correlation coefficient (r) was determined to be 0.972, with a *p*-value of 0.0000, indicating a highly significant positive linear correlation. This confirms a strong positive correlation between the CEUS outcome and the biopsy results.

## 4. Discussion

This retrospective investigation offers valuable insights into the efficacy of CEUS in assessing small renal lesions. The main aim of this study was to establish CEUS as a reliable method for characterizing kidney lesions, particularly when their nature, whether cystic or solid, is ambiguous in routine clinical practice and when their malignant or benign status is uncertain. Patients presenting with indeterminate small renal masses often necessitate additional imaging examinations, such as contrast-enhanced computed tomography (CT) and magnetic resonance imaging (MRI) [37]. Nevertheless, the sensitivity and specificity of distinguishing renal cell carcinoma (RCC) from other tumors using these methods have been documented to range from 73% to 100% and 84% to 91%, respectively [38].

The robust positive diagnostic accuracy coupled with the demonstrated high sensitivity and specificity of CEUS in this study affirm its reliability as a dependable imaging method for characterizing small renal lesions. CEUS displayed sensitivity in detecting malignant SRMs on par with that of CT and MRI (as reported at 97.1% and 96.4%, respectively, in a 2019 study by Marschner et al. [39]), suggesting its effectiveness in identifying potential malignancies within renal masses. Similarly, the specificity of CEUS in discerning benign SRMs was comparable to that of CT and MRI (as reported at 47.4% and 75%, respectively, in a 2019 study by Marschner et al. [39]), indicating its ability to accurately classify lesions as non-malignant. These findings underscore the potential of CEUS as an alternative imaging modality for evaluating SRMs. The comparable diagnostic accuracy of CEUS suggests that it can be a reliable choice, especially for patients who may be ineligible for contrast-enhanced CT or MRI due to contraindications or limited access [40,41]. This retrospective analysis has revealed that CEUS demonstrated a notable positive predictive value (PPV) of 95%, a commendable specificity of 94%, and a satisfactory sensitivity of 87.5% in determining the tumor characteristics of ambiguous renal lesions. Notably, the primary challenges in interpretation were encountered in cases of oncocytomas and atypical angiomyolipomas, a phenomenon that appears congruent with findings from analogous studies highlighting the intricate differentiation challenges stemming from shared imaging characteristics [42]. Our findings are comparable to those reported in the scientific literature and support the notion that CEUS demonstrates good diagnostic performance in distinguishing between benign and malignant SRMs. Furthermore, these results align with a previous study conducted by Shu-Ping Wei, who reported a sensitivity of 93.5%, a specificity of 68%, and a positive predictive value (PPV) of 91.6% [43]. As per a study conducted by Arash Najafi et al. [44], the sensitivity and specificity, respectively, were as follows: 98.1% and 95.4% overall, 100% and 95.6% for cystic lesions, and 97.7% and 90.6% for solid lesions. Tufano et al. have reported even more promising outcomes, with a sensitivity of 93%, specificity of 100%, and a positive predictive value (PPV) of 100%, as determined through the utilization of both qualitative and quantitative parameters [29]. Issues concerning the nephrotoxicity of contrast agents, radiation exposure risks, and high costs have also been raised. Recently, the development of new contrast media and imaging techniques has enabled contrast-enhanced ultrasound (CEUS), already actively used in organs like the liver [45]. CEUS allows the observation of both micro- and macrocirculation in renal masses, which may also aid in their diagnosis, despite differences in blood supply compared to liver masses. Moreover, ultrasound contrast agents have a lower incidence of side effects such as nephrotoxicity [33]. Patient-related factors, including meteorism and obesity, have been reported as the main factors negatively affecting CEUS image quality. While CEUS is primarily used for liver imaging, there has been a growing interest in renal applications of CEUS. Thus, determining which patient factors can reliably predict adequate CEUS image quality is of interest. However, recent developments in contrast media and imaging techniques have led to the emergence of CEUS as a promising alternative for the diagnosis and characterization of renal masses [46]. CEUS demonstrates high diagnostic performance and other advantages, including a low rate of side effects. Therefore, CEUS is generally preferred for characterizing focal renal lesions. A recent study even demonstrated the safety of CEUS in a small cohort of six pregnant women [47]. Knowing beforehand which imaging modality is likely to provide a diagnosis can expedite the diagnostic process, enhancing patient comfort and outcomes. By using CEUS only when diagnostic quality is expected, the immediate diagnosis can determine whether cross-sectional imaging is necessary for cancer staging in the event of a malignant lesion, thus avoiding unnecessary imaging in patients with benign lesions. In addition, CEUS has important economic implications, as it can help identify patients who do not need to undergo more expensive imaging methods such as MRI [48]. For example, in cases where an incidental renal mass is detected by plain B-mode ultrasound, physicians can use CEUS to predict whether CEUS or MRI might be the better imaging method for further characterization of the mass. This can help reduce healthcare costs and improve resource allocation. CEUS offers quick performance, with examinations typically lasting only a few minutes [4]. This efficiency is beneficial for both patients and healthcare providers, as it allows for efficient evaluation and immediate decision-making regarding further diagnostic or therapeutic interventions. The real-time imaging capabilities of CEUS enable clinicians to assess the perfusion patterns and enhancement characteristics of renal lesions, aiding in the differentiation between benign and malignant lesions and determining the specific type of lesion [49]. Another advantage of CEUS is that it can be less stressful for patients than other imaging modalities. Studies have shown that more than 50% of individuals experience high levels of anxiety during an MRI examination, which can lead to the occurrence of motion artifacts and other imaging errors. By contrast, CEUS is relatively non-invasive and can be performed quickly and easily in most cases [50]. Overall, therefore, CEUS represents a promising new tool in the diagnostic and treatment arsenal of physicians. By carefully selecting the right imaging modality for each patient and situation, physicians can achieve more accurate diagnoses, reduce patient discomfort, and improve outcomes [51]. CEUS offers several advantages over other imaging modalities for evaluating small renal masses. It provides real-time imaging capabilities without the associated risks of nephrotoxicity or radiation exposure, as seen with CT and MRI. CEUS has demonstrated high diagnostic performance and can offer valuable insights into clinical decision-making. Furthermore, CEUS is well-suited for long-term surveillance and follow-up in patients with renal cysts [35,47,52]. The field of medical imaging has advanced significantly in recent years, providing physicians with a range of modalities to choose from when diagnosing and treating patients. Yet not all imaging modalities are equally effective in all situations, and choosing the right one can be critical to achieving an accurate diagnosis and determining the best course of treatment [6]. However, certain limitations must be considered when employing CEUS, including patient-related characteristics such as obesity and meteorism, which can affect image quality. CEUS is also operator-dependent, with the radiologist’s experience and expertise playing a crucial role in image interpretation [53]. The measurement of a renal lesion is an important aspect of characterizing it accurately. It is important to note that the radiologist may have made an error in the ultrasound description, which could have a significant impact on the diagnosis. In addition to the potential for errors in the ultrasound description, there are also certain limits to this diagnostic technique. For example, ultrasound may not be able to provide a clear picture of the entire renal mass, which could limit its ability to accurately diagnose certain types of renal lesions [54,55]. Despite these limitations, contrast-enhanced ultrasound (CEUS) has emerged as a promising diagnostic tool for distinguishing between malignant and benign renal masses. The high degree of accuracy that CEUS provides can be invaluable in clinical decision-making, particularly in cases where localized renal masses are present. These advantages contribute to accurate diagnoses and aid in treatment decisions. This study had certain drawbacks. To begin with, it was a retrospective study, and secondly, the limited number of participants may constrain the ability to generalize the study’s findings. Furthermore, owing to the restricted quantity of renal masses encompassed in this study, an examination of variations within individual histotypes of renal masses was not undertaken. To conduct a more comprehensive investigation, it is imperative to have a larger sample size in future research. Such a study should aim to replicate this study’s methodology and results to ensure the generalizability and reliability of the findings.

## 5. Conclusions

The findings of this study provide insightful information concerning the accuracy and potential of CEUS in diagnosing and characterizing small renal masses. CEUS plays a significant role as a problem-solving technique for characterizing renal lesions. It is a promising additional diagnostic tool capable of distinguishing between malignant and benign renal masses. The high degree of diagnostic accuracy observed can influence the clinical decision-making strategy for localized renal masses. Future validation studies involving larger and more diverse patient populations are needed to confirm the diagnostic accuracy of CEUS. Continued investigation into the diagnostic accuracy and clinical utility of CEUS in larger patient populations will also help establish its role in routine clinical practice. Additionally, ongoing advancements in technology and imaging protocols may address some of the limitations associated with CEUS, further enhancing its diagnostic capabilities in the evaluation of small renal masses.

However, it is important to note that despite its safety and effectiveness, CEUS is not yet widely practiced by all radiologists. Efforts to promote education and training in CEUS will be crucial for increasing its adoption in clinical practice, ensuring that more patients benefit from this valuable diagnostic tool.

## Figures and Tables

**Table 1:** Study population characteristics regarding clinical information.

Characteristic	Mean	Minimum	Maximum
Age	62 years	37 years	87 years
Tumor Dimension	23.5 mm	10 mm	30 mm

**Table 2:** Study population characteristics regarding diagnostic pathway. CEMRI (contrast-enhanced magnetic resonance imaging); CECT (contrast-enhanced computed tomography).

Characteristic	Number of Patients (*n*)
Study population	44 patients
Small benign renal masses	21 patients
Small malignant renal masses	19 patients
Previous to CEUS diagnostic imaging pathway: - Non-contrast US + CEMRI - Non-contrast US + CECT - Non-contrast US + CECT + CEMRI	18 patients 21 patients 4 patients
- Patients referred to 3-month follow-up	19 patients
Patients undergoing histological assessment - Through Fine Needle Aspirated Biopsy - Through surgical excision	22 patients 6 patients 16 patients

## Data Availability

Data are available from the corresponding author.
